# Surgery

**Published:** 1907-09

**Authors:** T. Carwardine


### SURGERY.

There have been few surgical advances within recent years to compare, in their practical importance, with those made in the **treatment of general peritonitis.** A few years ago it was very exceptional for any patient to recover after a widely-diffused suppurative peritonitis; but the conditions are different nowadays, and by the application of modern methods of treatment a large proportion of patients may be saved, even in cases in which there is widely-generalised peritoneal inflammation. The two most important advances in treatment are those generally known in association with the names of Drs. Fowler and Murphy; and although they have only been in use the last few years, they have become recognised as standard methods. The Fowler position consists in placing the patient in the upright sitting posture as soon as possible at the time of, or immediately after, operation; so that the peritoneal exudation may gravitate to the lower abdomen, where drainage is provided for, away from the more dangerous diaphragmatic region. Dr. Murphy explained his methods before the American Medical Association in 1904. The principles are: (1) Rapid removal of the focus, and closure of the h le in the gut, with as little disturbance of the peritoneum as possible; (2) Drainage through the operation-wound and above the pubes; (3) Rapid procedure, preferably through the rectus, and without the insertion of sutures; (4) Food is withheld from the mouth for two or three days; (5) Continuous saline injections are given by the rectum. These are administered from an irrigator, placed from four to six inches above the level of the anus by means of tubing and a short nozzle having lateral openings. Thus from twelve to twenty pints may, under favourable conditions, be absorbed; and not only is the thirst relieved thereby, but the tongue becomes moist, there is an increase of the urinary and peritoneal flow, and the heart and kidneys are stimulated. The idea is that, by the continuous injection of saline, the direction of osmosis is reversed, and there is accordingly a free flow of peritoneal exudate through the wound.

Although these measures are generally recognised and acted upon, there are minor variations in detail employed by different surgeons, which it may be instructive to survey. In a paper read by Le Conte before the Philadelphia Academy of Surgery[1] and subsequently discussed, we have the following views. The chief factors in abdominal drainage through a pelvic tube are the effect of gravity and the pump-like action of the diaphragm. The rectal irrigation causes such an increase of peritoneal flow that the dressings become readily soaked, and there is an increase in the amount of urine passed. Dr. Gibbon had employed the Fowler position and Murphy treatment with success, but he had not been able to get his patients to absorb so much saline fluid as mentioned. Dr. Harte was convinced of the importance of keeping food away from patients after operation, thus materially diminishing the tendency to distension. The question of drainage was more especially considered in a paper read by Knott[2] before the Chicago Surgical Society and subsequently discussed, and in some respects his method presents some differences. He believes that free drainage of the infected peritoneum is the essential factor in successful treatment, and supports his argument by a record of seventeen recoveries out of nineteen cases, the fatal cases comprising one originating from the appendix, and the other from a large ruptured suppurating ovarian cyst. He thinks that the abdominal incision should always be ample, and made in the middle line either above or below the umbilicus, as the case may be. This is followed by immediate and thorough repair of the diseased parts. Then an incision is made just above the symphysis, and through this a large rubber tube at least one inch in

[1] *Ann. Surg.*, 1906, i. 231, 314. [2] *Ibid.*, 1905, ii. 75, 316.

diameter, previously split from end to end and carrying a gauze wick, is passed to the bottom of the pelvis. Abdomino-vaginal drainage may be similarly arranged for, using a rubber tube without gauze. The abdominal cavity is then thoroughly washed out with gallons of hot salt solution, care being taken to reach all the fossæ and concealed septic areas. The upper incision is then rapidly closed, but before tying the last stitch the abdomen is filled with salt solution by means of a funnel. The lower wound is left open, and in males an additional tube, similar to the first but without the wick, is introduced to the bottom of the pelvis alongside the first. The patient is then raised to the sitting posture while yet on the table, and this position is maintained during removal and subsequently when in bed. The dressings require frequent renewal, and when the flow becomes more scanty the fluid is pumped out through the plain tube every two hours for the next twenty-four hours. All tubes may be withdrawn in from five to eight days. He makes the incision a long one, and closes it except where the drain is inserted, and attaches great importance to filling the abdomen with salt solution ; and entirely avoids drainage with plain gauze. Since adopting this technique he states that his mortality has fallen from 90 per cent. to 11 per cent. He strongly objects to evisceration, partial or complete. In the discussion which followed the reading of this paper, Dr. Jacob Frank stated that the only two cases of diffused septic peritonitis that he had ever had recover in the last twenty years were treated on the lines advocated by Dr. Knott. Dr. Eisendrath had met with similar remarkable success by using the Fowler position, avoiding evisceration, but flushing copiously with saline. He pointed out the possibility of similar success by different methods, Dr. Murphy by non-irrigation saving fifteen out of sixteen, and Dr. Mordecai Price saved the whole of seventeen cases by flushing. Others remarked on the importance of not overlooking the right renal pouch ; and a heroic fact was mentioned, in which a surgeon reopened the abdomen of his own son eighteen hours after an operation by another surgeon, then absent, and by means of the treatment advocated by Dr. Knott saved the boy's life.

It is well known that after operations for generalised peritonitis there is often a considerable amount of distension or even intestinal obstruction. The surgeon is then in doubt as to whether anything further should be done. Dr. H. Lilenthal records a case in which, after operation following peritonitis with symptoms of obstruction, he reopened the abdomen above the umbilicus, let out a quantity of fluid, and performed a temporary enterostomy on a much-distended loop of jejunum, which he subsequently closed by a secondary suture, the patient recovering.[1] It may

[1] *Ann. Surg.*, 1905, i. 944.

be marked that the evidence is not clear that the enterostomy was the sole reason of success ; it may be that the liberation of the fluid was the more important factor in the relief of obstruction.

The subject was discussed at a meeting of the British Gynæcological Society,[1] when Mayo Robson recapitulated the lines of treatment already referred to. Differences of opinion were expressed, some recommending morphine and alcohol with free abdominal flushing, some free purgation by salines or calomel, and some the application of ice at the onset of peritonitis. It cannot be said that the subsequent discussion revealed full practical acquaintance with the advantages of modern treatment. The same may be said of a discussion on the subject at the Royal Academy of Medicine in Ireland.[2]

Before the Royal Medico-Chirurgical Society, Mr. Malcolm expressed views concerning inflammation similar to those previously expressed by Bantock, his view that peritonitis was due to irritation and the temperature simply a nervous reflex being met by arguments from Mr. Dudgeon, who showed that in even the so-called sterile exudate of peritonitis, micro-organisms chiefly staphylococcus albus, could be found.[3]

There are two forms of acute peritonitis, pneumococcal and puerperal, which demand attention ; the former in particular having come into special prominence of late. Dr. F. S. Mathews read a paper on " pneumococcal peritonitis," with a report of five cases.[4] He also refers to two papers on the same subject, one by Jensen comprising 106 cases,[5] and one by von Brüns.[6] From a consideration of these papers it is seen that pneumococcal peritonitis is three times as frequent in children as in adults, and that although the sexes are equally affected in adult life, in children it is seven times as frequent in girls as in boys. The peritoneal exudate is copious, thick, and odourless, with a tendency to localisation. The majority of cases have been primary in the peritoneum, and some have been associated with or followed by pneumonia, empyema, pericarditis, otitis media or intestinal lesions. The most characteristic symptoms are : (1) Sudden onset with fever ; vomiting for a day or two ; slight pain, tenderness and distension ; little muscular rigidity. This is followed by an improvement in the symptoms.[5] (2) Then, with the increase of exudate, there appears a tense cystic mass in the hypogastrium, the temperature rises and becomes of remittent type, succeeded by cachexia, weakness, followed by death or recovery after operation. The localised form is more amenable to successful operation, 80 per cent. being successful. The differential diagnosis is difficult,

[1] *Brit. M. J.*, 1907, i. 1483. [2] *Ibid.*, 1906, i. 862.
[3] *Ibid.*, 1905, ii. 1116. [4] *Ann. Surg.*, 1904, ii. 698.
[5] Jensen, *Arch. f. klin. Chir.*, 1903, lxx. 91.
[6] Von Brüns, *Beit. z. klin. Chir.*, 1903, xxix. 57.

and no definite distinguishing point can be stated. The modes of pneumococcal infection of the peritoneum are as follows : through a wound, through the diaphragm, through the genitals, through the intestinal tract, through the blood, and from pneumococcic foci in abdominal organs. It is interesting, however, to notice that in cases of pneumonia, pneumococci may be present in the peritoneum without causing peritonitis.

With regard to puerperal general peritonitis, one may refer to a paper by Dr. Ellice McDonald on the subject.[1] Of eleven cases under his care, four were associated with streptococci, two with staphylococci, one with pneumococci, one with gonococci, and three with multiple infections of the streptococcus associated with the bacillus coli, gonococcus, or the bacillus aërogenes capsulatus. It will thus be seen that puerperal peritonitis is usually streptococcic and usually fatal, though not invariably so. The staphylococcus aureus has been rarely found, and although the gonococcus is such a frequent cause of salpingitis, it is not so commonly the cause of puerperal peritonitis. For further details and references to this subject the paper may be consulted ; but with regard to the surgical aspects of the question, there seems to be evidence that polyvalent anti-streptococcic serum may be of some utility, used in association with operation after the method of Murphy for peritonitis in general. That the results are improving is shown by a collection of 121 cases by Jeannin, with nearly 50 per cent. of recoveries.

T. CARWARDINE.

